# Supplementary material for: Structural Characterization and Functional Analysis of Mevalonate Kinase from Tribolium castaneum (Red Flour Beetle)
Source: Int J Mol Sci. 2024 Feb 22;25(5):2552. doi: 10.3390/ijms25052552 (PMC10931659; doi:10.3390/ijms25052552)
Supplement: Supplementary file 1 [file ijms-25-02552-s001.zip › ijms-2833561-supplementary.pdf]

Supplementary Materials

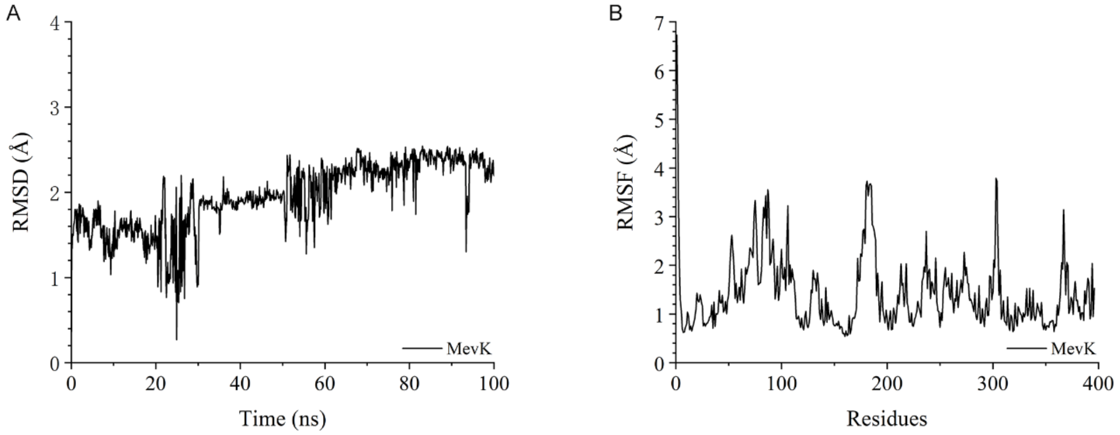

Figure S1. Stability checking of the *Tc*MevK model with molecular dynamics simulation. (**A**) The root mean square deviation (RMSD) to monitor the MD simulation for 100 ns. (**B**) RMSD plots showed the TcMevK stabilized at ~2.2 Å after 50 ns.

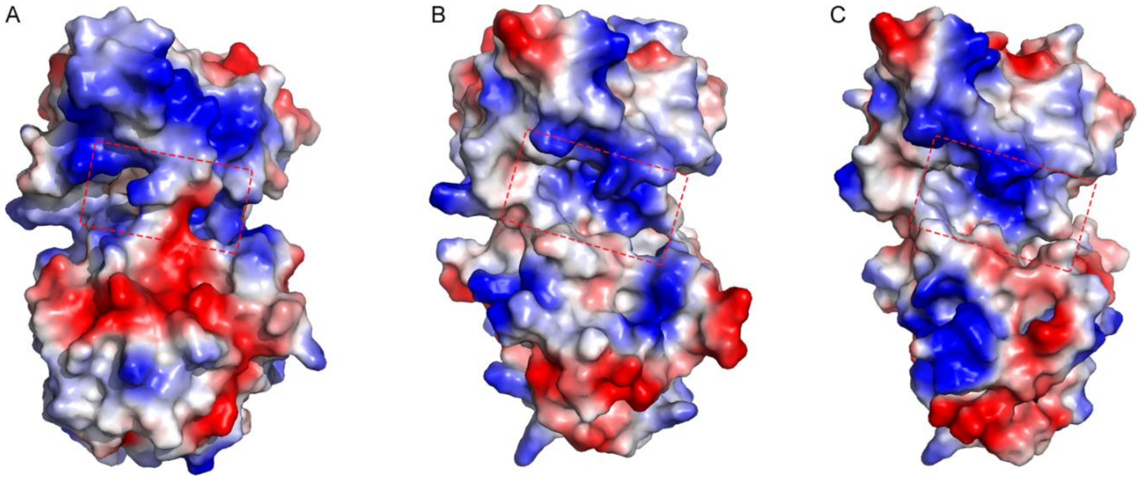

Figure S2. Surface representation of the *Tc*MevK (**A**), *Homo sapiens* MevK (**B**), and *Rattus norvegicus* MevK (**C**) to show the substrate-binding pocket. The substrate-binding pocket labeled as red rectangle box.

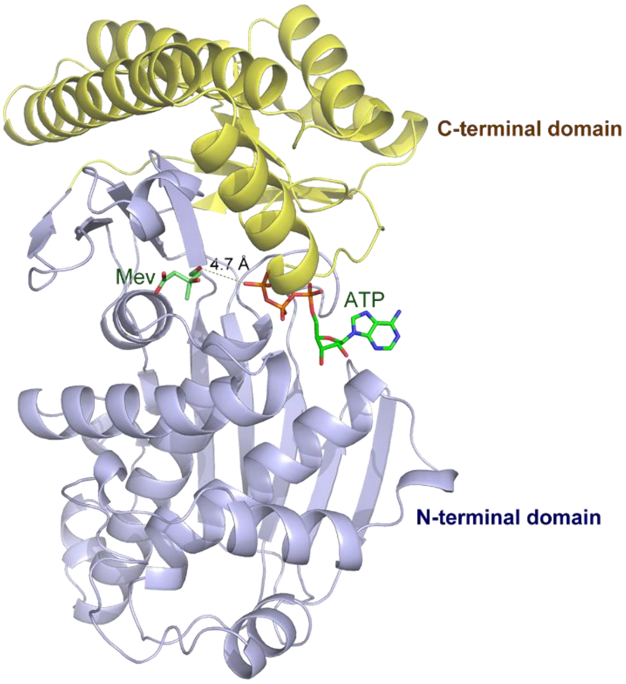

Figure S3. Cartoon representation of *Tc*MevK binding with and substrate Mev and cofactor ATP. .

Table S1. List of primers for quantitative RT-PCR.

Primer	Sequence (5′ to 3′)	Length (bp)
TcMevK-F	ATTGGACCAAGCTTGCGAGA	162
TcMevK-R	GTTAAAACCGCCCCGAAACC	
TcKr-h1-F	ACACCGTTGAATGCTGGTGA	181
TcKr-h1-R	TCCTTGGCGGAAGACTCAAC	
TcRps3-F	ACCGTCGTATTCGTGAATTGAC	186
TcRps3-R	ACCTCAAAACACCATAGCAAGC	
